# *Aquirufa esocilacus* sp. nov., *Aquirufa originis* sp. nov., *Aquirufa avitistagni*, and *Aquirufa echingensis* sp. nov. discovered in small freshwater habitats in Austria during a citizen science project

**DOI:** 10.1007/s00203-025-04275-6

**Published:** 2025-02-25

**Authors:** Alexandra Pitt, Stefan Lienbacher, Johanna Schmidt, Meina Neumann-Schaal, Jacqueline Wolf, Aharon Oren, Sophia Reichl, Martin W. Hahn

**Affiliations:** 1 Research Department for Limnology, Universität Innsbruck, Mondsee, 5310 Mondsee, Austria; 2 Metabolomics and Services, Leibniz Institute DSMZ-German Collection of Microorganisms and Cell Cultures GmbH, Braunschweig, Germany; 3 The Institute of Life Sciences, The Hebrew University of Jerusalem, The Edmond J. Safra Campus, 9190401 Jerusalem, Israel

**Keywords:** *Aquirufa*, *Spirosomataceae*, Freshwater bacteria, Genome, Genome size, Citizen science

## Abstract

Within a citizen science project, 112 freshwater habitats in Austria were sampled to get bacterial cultures belonging to the genus *Aquirufa* using a strategy for targeted isolation. We focused on these bacteria because they are widespread and represent typical freshwater bacteria and, furthermore, the typic red pigmentation facilitates preselection. Among the 113 obtained *Aquirufa* strains were HETE-83D^T^, KTFRIE-69F^T^, OSTEICH-129V^T^ and PLAD-142S6K^T^, originating from small ponds and a creek. Phylogenetic reconstructions with 16S rRNA gene sequences and genome-based analyses with amino acid sequences of 501 core genes showed that all four strains belonged to the *A. antheringensis* branch of the genus *Aquirufa*. Calculation of whole-genome average nucleotide identity values and digital DNA-DNA hybridization values revealed that they represent in each case a new species. The genome sizes of the four strains were between 2.5 and 2.8 Mbp and the G + C values were between 41.4 and 41.8%. Like all type strains of the genus *Aquirufa*, cells were rod-shaped, and liquid cultures and colonies on agar plates were red-pigmented, likely due to carotenoids. All strains except OSTEICH-129V^T^ showed gliding motility on soft agar plates. All strains grew aerobically but only PLAD-142S6K^T^ could grow weakly under anaerobic conditions. We propose here to establish the names *Aquirufa esocilacus* sp. nov. for strain HETE-83D^T^ (= DSM 118087^T^ = JCM 37094^T^), *Aquirufa originis* sp. nov. for KTFRIE-69F^T^ (= DSM 117798^T^ = JCM 37095^T^), *Aquirufa avitistagni* for OSTEICH-129V^T^ (= DSM 118088^T^ = JCM 37100^T^) and *Aquirufa echingensis* sp. nov. for PLAD-142S6K^T^ (= DSM 117799^T^ = JCM 37096^T^).

**Supplementary Information:**

The online version contains supplementary material available at 10.1007/s00203-025-04275-6.

## Introduction

The genus *Aquirufa*, belonging to the phylum *Bacteroidota*, was established in 2019 (Pitt et al. 2019), at that time assigned to the family *Cytophagaceae* and reclassified in 2024 to the family *Spirosomataceae* (Pitt et al. 2024). Lu et al. suggested splitting the family *Spirosomataceae* and establishing a new family, named *Flectobacillaceae*, including the genera *Aquirufa*, *Sandaracinomonas*, *Flectobacillus*, *Arcicella* and *Pseudarcicella* (Lu et al. 2024). At the time of writing (September 2024) the genus *Aquirufa* comprised eight species with validly published names (Pitt et al. 2019, 2020, 2022, 2024; Oren and Garrity 2022; Oren and Göker 2024; Sheu et al. 2020). All species have in common that the associated strains were isolated from various freshwater habitats. Common features of these bacteria are rod-shaped cell morphologies, gliding motility, red pigmentation, and aerobic and chemoorganotrophic metabolism. Menaquinone-7 occurs as major menaquinone and phosphatidylethanolamine is the major identified polar lipid. The genome sizes of the type strains of the genus range from 2.5 to 3.2 Mbp and the G + C values from 38.0 to 42.6%. The genus is phylogenetically split into two distinct lineages, the *A. antheringensis* branch and the *A. nivalisilvae* branch, which differ in genome sizes and G + C values (Pitt et al. 2024). Species of the *A. antheringensis* branch show genome sizes of about 2.5 Mbp and G + C contents of about 42%, in contrast, the values for species of the *A. nivalisilvae* branch are about 3.0 Mbp and 38%, respectively.

Since bacteria belonging to the genus *Aquirufa* are widespread in freshwaters worldwide and occur in some habitats in high abundance (Pitt et al. 2024), they seem to constitute an important group of freshwater bacteria. The 16S rRNA gene sequence similarities between the species within the two *Aquirufa* branches are very high (99.3 to 100%), so it is impossible to differentiate species based on the 16S rRNA gene as a marker. This means that available data from environmental amplicon sequencing studies do not help to explore the particular environmental importance of *Aquirufa* species.

Within our citizen science project, we aimed to learn more about the biodiversity, distribution and ecology of the genus *Aquirufa*. We focused on aquatic systems in a geographically limited area of Austria but tried to incorporate a wide range of freshwater habitat types. In the first part of the project, we endeavored to cultivate bacterial isolates belonging to the genus *Aquirufa*. Since the characteristic red pigmentation facilitates the search, many pure cultures of strains belonging to the genus could be established. Several strains were genome-sequenced and subsequent calculation of whole genome average nucleotide identity (gANI) values suggested that some isolates represent new species (see below ‘Evidence for four new species’). After the first description of a new *Aquirufa* species, *Aquirufa regiilacus* (Pitt et al. 2024), originating from the current citizen science project, we present here the description of four new species, *Aquirufa esocilacus* sp. nov., *Aquirufa originis* sp. nov., *Aquirufa avitistagni* sp. nov., and *Aquirufa echingensis* sp. nov. with HETE-83D^T^, KTFRIE-69F^T^, OSTEICH-129V^T^ and PLAD-142S6K^T^, respectively as the type strain.

## Materials and methods

### Sampling and isolation of strains

Students of six school classes, members of the Global Underwater Explorers Austria and additional citizen scientists sampled various aquatic habitats and conducted measurements of environmental parameters. All samples involved in this study originated from Austria. The citizen scientists were encouraged to take water samples from various freshwater habitats. Measurements of pH values were performed using various pH-test strips (Dosatest^®^) and conductivity with a compact conductivity meter (LAQUAtwin-EC-11; HORIBA). Some habitats were visited once again to verify the measurements with a WTW multiparameter probe. The water samples were filtered through 0.65 µm pore size filters to exclude larger cells and the filtrates were spread on nutrient broth-soytone-yeast extract (NSY) agar plates (Hahn et al. 2004). The citizen scientists worked thereby under the supervision of the scientists. The agar plates were transferred to the laboratory and after incubation (21 °C, daylight, aerobic) for 10 days they were screened for colonies with the characteristic red pigmentation of *Aquirufa* cultures. Such colonies were transferred to a liquid medium and were purified by four times alternating cultivation on NSY agar plates and in NSY liquid medium. To assign isolates to the genus *Aquirufa*, and if possible to a known species, partial sequences of the marker gene *gyrB* encoding the B subunit of the DNA gyrase (Pitt et al. 2022) were used. Nevertheless, in some cases, especially for some strains belonging to the *A. antheringensis* branch, the phylogenetic resolution of the *gyrB* gene is insufficient for species-specific identification, especially for sequence similarity values around 95% (see Fig. S1). Therefore, some of the strains, as well as strains likely representing a new species (similarity ≤ 92%, Fig. S1), were selected for genome sequencing and calculation of gANI values (see below). All strains were stored at -80 °C in a liquid NSY medium supplemented with 15% glycerol.

We used the QGIS Geographic Information System (http://www.qgis.org) to map the sampling sites and to plot the obtained isolates R (RCoreTeam 2020) and RStudio (version 4.2.2, package zoo) (RStudioTeam 2019). Some citizen scientists were later involved in the phenotypic investigations of the obtained strains and other citizen scientists created species names.

### Phenotypic and chemotypic characterisation

The phenotypic and chemotypic characterisation was conducted with the same set of investigations (Pitt et al. 2024) and methods (Pitt et al. 2022) as reported previously. In brief, potential anaerobic growth, the temperature range of growth, and tolerance for NaCl were tested on NSY agar plates inoculated with 100 µl of a well-growing liquid culture. For testing anaerobic growth, an anaerobic jar, where the oxygen of the air was removed with crystalline silicic acid (Microbiology Anaerocult^®^ A, Merck KGaA) was used. For all experiments inoculated agar plates were incubated under standard conditions in parallel and served for classification growth positive or weak, respectively. Temperatures were tested under aerobic conditions starting at 5 °C and increasing in at least 1 °C steps until the strains showed no growth. The same procedure served for testing NaCl tolerance by using steps of 0.1% w/v. For testing the motility of the strains, soft agar plates (0.1 g l^−1^ K_2_HPO_4,_ 1 g l^−1^ yeast extract, and 2.0 g l^−1^ agar) were inoculated with a drop of culture and cultured under aerobic conditions at 21 °C for fourteen days. Spreading over the whole agar plate within two weeks was regarded as positive. Cell shapes and metrics were determined with an epifluorescence microscope (UV filter) after fixing liquid cultures with 2% paraformaldehyde and staining by 4′,6-diamidino-2-phenylindole (DAPI). The compounds of the fatty acids, polar lipids and respiratory quinones of the four strains were determined. For these purposes, the biomass of liquid cultures (NSY medium, 21 °C, three days incubation, 500 ml Erlenmeyer flasks, 250 ml culture, horizontal shaker, 90 min^−1^) was collected by centrifugation. For the cellular fatty acid composition, the biomass (11–12 mg dry weight) was saponified and methylated followed by analysis on an Agilent Technologies 6890 N instrument coupled to a flame ionization detector as described in the protocol of Sasser (Sasser 1990). Equivalent chain length values were calculated according to the Microbial Identification System Sherlock to provide the peak naming according to the TSBA6 database. In addition, we performed analyses of the extract by gas chromatography/mass spectrometry (GC/MS) on an Agilent Technologies 7000D GC/MS Triple Quad instrument, to identify the fatty acids precisely (Vieira et al. 2021). If double bond positions occurred, they were identified by further derivatization to dimethyl disulfide adducts and subsequent GC/MS analysis (Moss and Lambert-Fair 1989). The polar lipids were extracted and analyzed according to the methods of Tindall (Tindall 1990a, 1990b), which were based on the description by Bligh and Dyer (Bligh and Dyer 1959). For segregation of the polar lipids two-dimensional silica gel thin-layer chromatography was used. The total lipids were detected by the usage of dodecamolybdophosphoric acid (Dmp). In addition, specific functional groups were detected by the usage of α-naphthol, ninhydrin, and molybdenum blue. Respiratory quinones were analyzed as described previously (Vieira et al. 2021). Briefly, 10–13 mg biomass was resuspended in 0.75 ml hexane/methanol (1:2, v/v) and stirred at ambient temperature for 10 min. 0.25 ml hexane was added, mixed and the non-polar phase was collected. The polar phase was re-extracted with hexane and the combined non-polar phase were loaded onto a Silica Chromabond solid phase extraction column (Macherey–Nagel, Düren, Germany), pre-equilibrated with hexane. The column was washed 3 times with hexane and the respiratory quinones were eluted with 10% methyl-tert-butyl-ether in hexane. Extracts were dried under a stream of nitrogen, reconstituted in methanol and analyzed via HPLC on a Nucleosil 120–3 C18 column (Macherey–Nagel, Düren, Germany) with methanol as solvent using a 1260 Infinity II HPLC system, coupled to a diode array detector (Agilent Technologies, Waldbronn, Germany). Mequinones were identified by absorption spectrum and retention time. Extracts of known composition were analyzed in the same run. A solid-phase extraction protocol was used for quinone extraction, and the analysis was performed by reversed-phase HPLC coupled to a diode array detector using an Agilent Technologies 1290 Infinity II system.

### Genomic characterisation

DNA extraction and genome sequencing were performed following the method described previously (Hoetzinger et al. 2017). 200 ng Qubit quantified genomic DNA was used for a shotgun library which was paired-end sequenced on an Illumina NovaSeq sequencer with 2 × 150 bp. Genomes were de novo assembled by SPAdes version 3.13.1 (Bankevich et al. 2012) and checked for quality by CheckM lineage_wf (Parks et al. 2015), provided by The European Galaxy server (Galaxy-Community 2024). The genome sequences of the strains described here were annotated by the NCBI Prokaryotic Genome Annotation Pipeline (Tatusova et al. 2016) and deposited at DDBJ/ENA/GenBank databases. In addition, the genomes were annotated by the RAST annotation server (Aziz et al. 2008) and the Integrated Microbial Genomes & Microbiomes Expert Review (IMG/MER) system (Chen et al. 2019) and included in the IMG/MER database. The IMG/MER ID numbers of the new genomes and the genomes used for comparison are shown in Table S1. The IMG/MER system served also for calculating gANI values for all possible pairs including the new and all *Aquirufa* type strains of the *A. antheringensis* branch, digital DNA-DNA hybridization (dDDH) values were calculated with the Type (Strain) Genome Server (Meier-Kolthoff et al. 2021) with formula d_4_.

### Phylogenetic reconstructions

For phylogenetic reconstructions, we chose all type strains of *Aquirufa* species and type strains of the nearest affiliated genera *Sandaracinomonas*, *Pseudarcicella*, *Arcicella* and *Flectobacillus* with available genome sequences. A phylogenetic tree based on almost full-length 16S rRNA sequences was calculated using the software Mega X (Kumar et al. 2018). All considered sequences were aligned and subsequently trimmed. The dataset served for the construction of a neighbour-joining tree with 1000 replicates according to the Kimura 2 parameter model (Kimura 1980), with the settings gamma-distributed (1 gamma parameter) and gaps pairwise deleted. A genome-based phylogenetic tree was calculated with amino acid sequences of the core genomes. For this, all used genome sequences were annotated by Prokka version 1.14.6 (Seemann 2014) with the standard settings. The received gff3-files served for the identification of the core genes of the pan-genome using Roary version 3.13.0 (Page et al. 2015). The setting for considering a gene as a core gene of 70% minimum sequence similarity for blastp and the requirement that a gene has to occur in 100% of all genomes resulted in the identification of 501 core genes. For both tools, we utilised The European Galaxy server (Galaxy-Community 2024). The obtained nucleotide sequences were translated into protein sequences with Mega X (Kumar et al. 2018) and aligned by MAFFT (Katoh et al. 2005). The tool Gblocks, version 0.91b (Castresana 2000) was used to filter out highly variable positions of the obtained alignment. The primary alignment was thus reduced from 187,353 to 172,414 positions (92%) in 817 selected blocks. A RAxML tree (Stamatakis 2014) was constructed using the platform CIPRES Science Gateway version 3.3 (Miller et al. 2010), with standard settings and 100 bootstrap replicates.

### Analyses of publicly available metagenomes

123 publicly available metagenomes obtained from freshwater samples worldwide were analyzed by mapping as described previously (Hahn et al. 2022; Hoetzinger et al. 2024). For this purpose, we used a set of concatenated genome sequences of all twelve *Aquirufa* type strains including the new ones with all ribosomal operons removed. On the European Galaxy server (Galaxy-Community 2024), the reads of the metagenomes were mapped on the concatenated set of the 12 genomes using Bowtie2 (Langmead and Salzberg 2012) with the setting of a 95% sequence similarity threshold reflecting the species demarcation threshold. The coverage of every base of the genome set was obtained using Bedtools (Quinlan and Hall 2010). The data were analyzed using R (RCoreTeam 2020) and RStudio (RStudioTeam 2019). We considered the detection of a particular species if the mapping on the genome of its type strain resulted in a coverage breadth of more than 50%. Lower coverage breadth combined with low coverage depth, potentially indicating that the number of sequenced base pairs limited the detection of the reference genome, could also be evidence for the occurrence of a species. So, we used the following analysis to create a test criterion for distinguishing between detection and no detection in cases of coverage breadth < 50% of genome size. Metagenomes harboring genomes of *Polynucleobacter paneuropaeus* (i.e., mapping results with coverage depth > 100-fold and breadth > 70%) were separately mapped on genome sequences of five *Polynucleobacter paneuropaeus* strains (ANI > 98% but differing in their sets of accessory genes). The number of mapped metagenomic reads was stepwise reduced and the respective coverage depth and breadth were recorded. The plotted results showed for all five *Polynucleobacter paneuropaeus* genomes asymptotic curves (Fig. S2). The genome-specific data set with the smallest asymptote (reflecting the size of the core genome) was used to model the curve. The obtained function Breadth-Threshold = -255.58 * 0.358^CoverageDepth + 75.27 was used to test whether a certain mapping result must be considered as detection or no detection. If the coverage breadth obtained by mapping metagenomic reads is at least tenfold and is larger than the calculated Breadth-Threshold, a particular species is supposed to be detected.

## Results and discussion

### Habitats and isolated strains

**Fig. 1:**
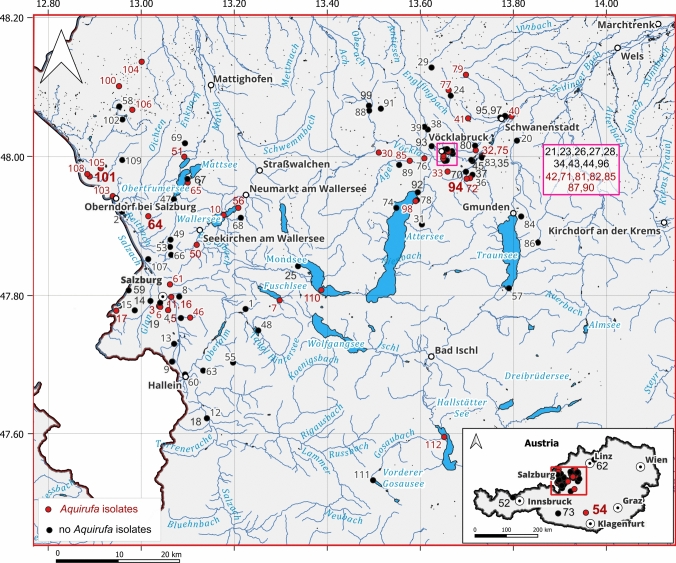
Map with all sampled freshwater habitats. The habitat numbers refer to Table S2. Red numbers: habitats from which *Aquirufa* strains could be isolated, black numbers: no *Aquirufa* isolates obtained, bold red numbers: home habitats of new isolates, strains HETE-83D^T^ from 64; KTFRIE-69F^T^ from 54; OSTEICH-129V^T^ from 94; PLAD-142S6K.^T^ from 101

**Fig. 2:**
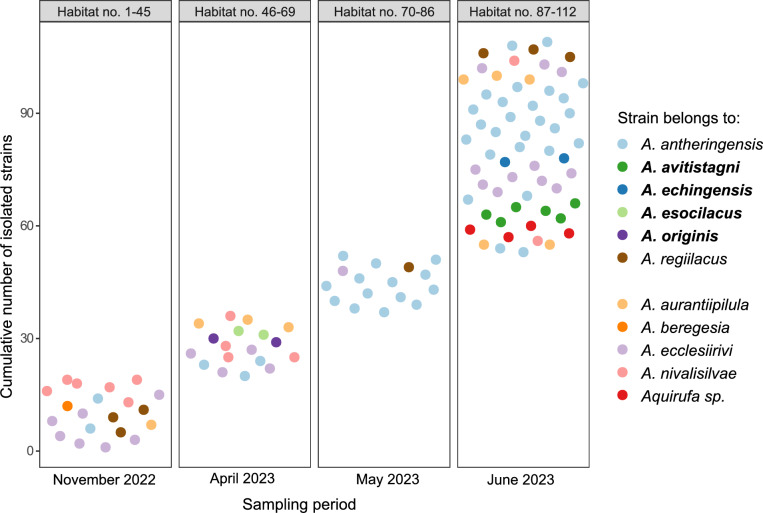
Cumulative number of strains of the genus *Aquirufa* obtained from 112 water samples (sampled habitats Fig. 1 and Table S2). Each dot represents a particular strain and the taxonomic assignments of the strains are indicated by different colors referring to a certain species. Species described here are in bold letters

Figure 1 displays all 112 aquatic habitats in Austria (Table S2), sampled in November 2022, April, May, and June 2023, and depicts the sampling sites where cultures belonging to the genus *Aquirufa* were obtained. The efforts in culturing *Aquirufa* strains were successful for almost 40% of the water samples (Table S2) in total, 113 pure cultures belonging to six described and five undescribed species could be obtained (Fig. 2). These data may include clonal and non-clonal cultures, as it was impossible to perform genome sequencing for all strains. Nevertheless, Fig. 2 represents the distribution of obtained cultures regarding the species affiliation. Interestingly, some species were overrepresented, especially *A. antheringensis* (Fig. 2), while strains of *A. lenticrescens* were not cultured. Each of the four new species described here was obtained only from a single habitat (Table S2). Only strains belonging to a single *Aquirufa* species could be cultured from most of the habitats while sampling of three habitats resulted in pure cultures representing three or four *Aquirufa* species (Table S2). These data and graphs illustrated two interesting points. First, although *Aquirufa* isolates typically grow well under standard laboratory conditions, numerous samplings and culturing efforts are necessary to obtain strains representing new species. Second, incorporating a broad diversity of sampled freshwater habitats, including habitats, like garden ponds, small creeks and ponds, resulted in the discovery of new species.

All four new strains described here were isolated from small freshwater habitats (Table S2, bold numbers). Strain HETE-83D^T^ originated from a small pond in Gauesed, Obertrum, Salzburg at the geographical coordinates 47.914312 N 13.015339 E. This pond has an area of approximately 500 m^2^ and some years ago a population of pikes lived there, while a few trout were added later. The water sample was taken in April 2023, and measurements in August 2024 revealed a pH of 7.3 and conductivity of 257 µS/cm. Strain KTFRIE-69F^T^ originated from a small artificial pond in Gloednitz, Carinthia at the geographical coordinates 46.894944 N 14.123163 E. The pond has existed for over 100 years and has an area of approximately 800 m^2^ with a maximum/average depth of 1 m. Formerly, the pond was used to produce electricity, but it is currently stocked for fishing trout and char once a year. The pond is drained in autumn and filled with water from a spring and a creek in spring. In April 2023, at the time of sampling the pH was 5.5 and the conductivity 64 µS/cm. Strain OSTEICH-129V^T^ originated from a small garden pond in Weiding, Rutzenmoos, Upper Austria at the geographical coordinates 47.968330 N 13.699821 E. The water sample was taken in June 2023. Measurements in July 2024 revealed a pH of 8.8 and a conductivity of 124 µS/cm. Strain PLAD-142S6K^T^ originated at the geographical coordinates 47.971592 N 12.889386 E from the creek Pladenbach in St. Georgen near Salzburg. This creek flows via the creek Moosach into the river Salzach. The yellowish water sample was taken in June 2023 and measurements in August 2024 revealed a pH of 8.3 and a conductivity of 490 µS/cm.

### Phenotypic and chemotypic characteristics of the strains representing new species

The phenotypic and chemotypic characteristics of the new strains are shown in Table 1, the fatty acid compositions are presented in Table S3 and the patterns of the polar lipids in Fig. S3. For most features, the new strains differed only slightly from the nearest related type strains of the genus *Aquirufa* (Table 1). Nevertheless, OSTEICH-129V^T^ differed from all other strains in growth behavior in a liquid medium. While the other strains appeared as a red–orange suspension, liquid cultures of OSTEICH-129V^T^ contained, in addition, tiny orange globules (Table 1). This tendency of the cells to form clumps under laboratory conditions could also be seen in the microscopic images, as the cells formed small aggregates. PLAD-142S6K^T^ differed from the other type strains by the ability to grow weakly in the anaerobic chamber on NSY agar plates (Table 1).

**Table 1:** Features of the type strains of the novel species and the nearly related species

	**1**	**2**	**3**	**4**	5	6	7
Mean cell length (µm)	1.10	1.10	1.38	1.60	1.70	1.20	0.80
Mean cell width (µm)	0.40	0.42	0.40	0.45	0.60	0.50	0.30
Liquid culture	red-orange suspension	red-orange suspension	red-orange suspension with tiny orange globules	red-orange suspension	red-orange suspension	red-orange suspension	red-orange suspension
Temperature range for growth (°C)	5–31	5–31 (w)	5–30	5–32	5–32 (w)	5–31 (w)	5–32 (w)
NaCl tolerance (%, w/v)	0	0	0–0.1	0–0.2	0–0.3 (w)	0–0.1 (w)	0–0.3
Gliding on soft agar	+	+	–	+	+	+	+
Anaerobic growth on NSY agar plates	–	–	–	w	−	−	−
*Respiratory quinones (% of the total content):*							
Menaquinone-7	97.1	96.5	92.6	95.4	≥ 99.0	99.4	94.0
Menaquinone-6	2.1	3.1	6.8	4.2	< 1.0	-	6.0
Menaquinone-8	0.8	0.4	0.6	0.4	-	0.6	-
*Major fatty acids (% of the total content)*:							
C_16:1_*ω*5c	11.1	7.2	7.9	5.3	11.9	7.0	9.1
C_16:1_*ω*7c	10.6	11.7	18.4	12.8	24.7*	12.0*	16.8
iso-C_15:0_	40.2	45.2	34.3	28.8	20.3	41.4	31.0
anteiso-C_15:0_	14.4	10.2	14.0	13.0	5.6	15.5	11.0
iso-C_15:0_3-OH	3.6	7.2	6.7	7.1	13.4	4.5	3.7
*Number of polar lipids:*							
Unidentified aminolipids	–	–	–	−	−	−	1
Unidentified phospholipids	1	1	–	−	−	−	−
Unidentified aminophospholipids	3	3	1	3	2	3	1
Unidentified glycolipids	2	2	1	1	−	−	−
Unidentified polar lipids	7	7	2	3	4	2	3

^1^*A. esocilacus* sp. nov. HETE-83D^T^; ^2^*A. originis* sp. nov. KTFRIE-69F^T^; ^3^*A. avitistagni* sp. nov. OSTEICH-129V^T^; ^4^*A. echingensis* sp. nov. PLAD-142S6K^T^; ^5^*A. antheringensis* 30S-ANTBAC^T^; ^6^*A. lenticrescens* 9H-EGSE^T^; ^7^*A. regiilacus* LEOWEIH-7C^T^

All strains had in common: cell morphology: rods, pigmentation colonies: red; identified polar lipid: phosphatidylethanolamine. Only the major fatty acids (≥ 10% for at least one strain) are listed, the whole fatty acid composition of the new strains is listed in Table S3. *and/or iso-C_15:0_2-OH; − negative; + positive; *w* weak (in brackets refers to the highest value)

All data were elevated in the same laboratories under the same conditions. Data from columns 5–7 were published previously (Pitt et al. 2019, 2022, 2024)

### Phylogeny of the strains representing new species

**Fig. 3:**
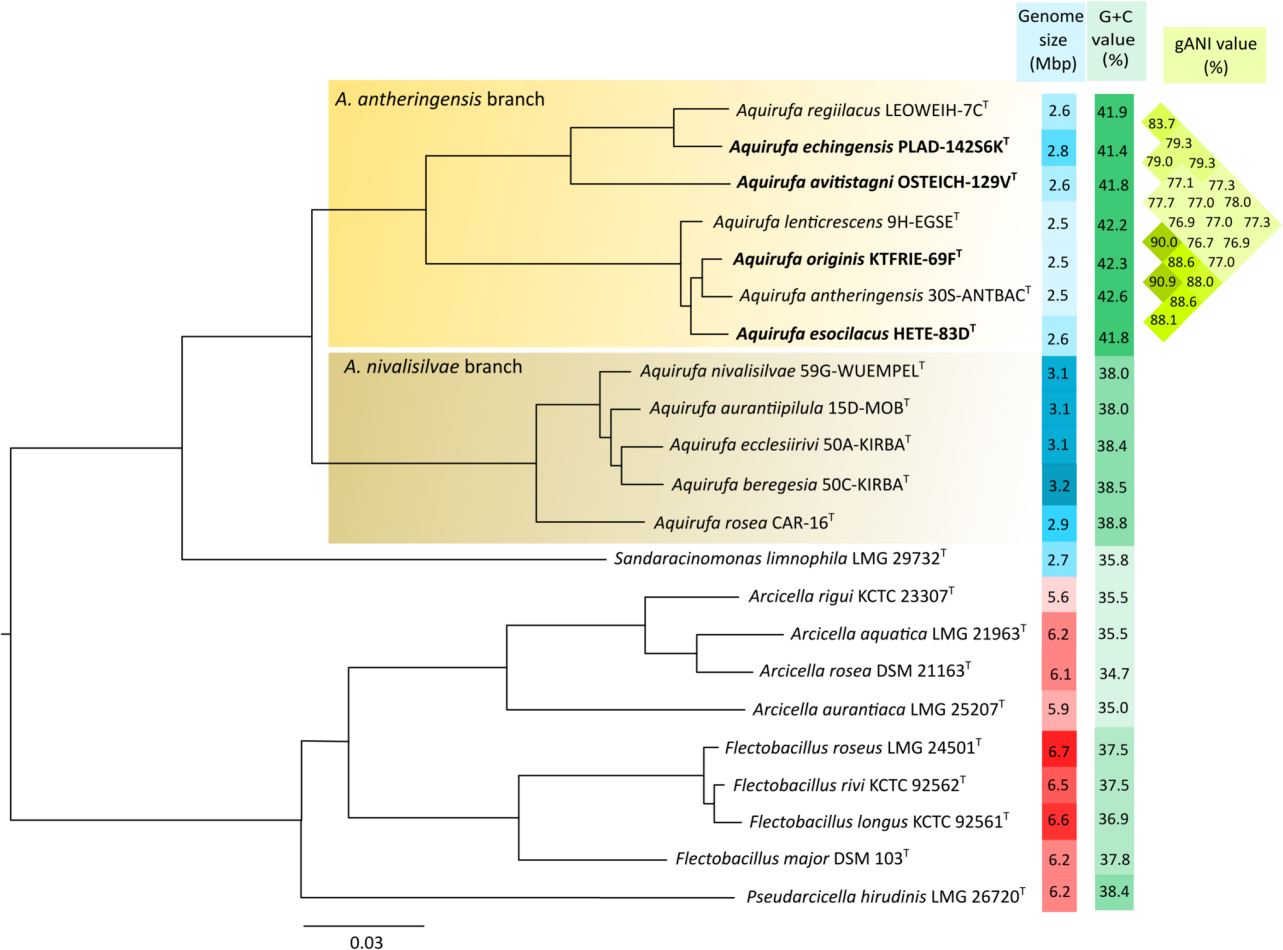
Reconstruction of the phylogenetic position of the new type strains and all previously described *Aquirufa* type strains and type strains of the closely related genera. A midpoint-rooted RaxML tree is shown based on the amino acid sequences of 501 core genes after filtering with Gblocks (see text). All accession numbers of the used genome sequences can be found in Table S1. Bootstrap values for all nodes 100%. The columns at the right depict the size and the G + C values of the genomes of the type strains and the pairwise genomic gANI values among the *Aquirufa* type strains of the *A. antheringensis* branch. Species described here are in bold letters

The phylogenetic reconstructions based on almost full-length 16S rRNA gene sequences (Fig. S4) showed that the four new strains belonged to the *A. antheringensis* branch of the genus *Aquirufa*. Pairwise 16S rRNA similarity values within this branch ranged from 99.3 to 100% (Table S4) and were above thresholds, for example, 98.7% (Chun et al. 2018), which are used for species demarcation. High inter-species 16S rRNA gene similarity values are well-known from the genus *Aquirufa* (Pitt et al. 2024) and other genera, for example, *Polynucleobacter* (Hahn et al. 2016). Therefore, the 16S rRNA sequence-based tree could not resolve the phylogenetic position of the new strains. The phylogenetic reconstructions based on whole genome sequences (Fig. 3) showed a similar structure of the *A. antheringensis* branch but revealed insights into the phylogenetic distances between the species of the branch. The genome-based tree pointed out that *Aquirufa antheringensis* and *Aquirufa lenticrescens* formed a cluster together with the two new strains KTFRIE-69F^T^ and HETE-83D^T^ with relatively small inter-species phylogenetic distances, which was also reflected by comparatively high gANI values of 88.0–90.9% (Fig. 3). The genome-based tree placed the new strain PLAD-142S6K^T^ near the type strain of *Aquirufa regiilacus* and OSTEICH-129V^T^ on a long branch beside them. The addition of the four new strains into the genome-based phylogenetic tree confirmed the impression that the species of the genus *Aquirufa* are separated on two distinct branches (Fig. 3).

### Genomic characteristics of the strains representing new species

All data concerning the assembled draft genomes of the four new strains are presented in Table S5, the genome sizes and G + C values are presented in addition in Fig. 3 in comparison with all *Aquirufa* type strains and species of related genera. The authenticities of the draft genomes were confirmed by comparison with Sanger sequences of the 16S rRNA gene. All four genomes included three ribosomal RNA operons. Interestingly, the genome size of strain PLAD-142S6K^T^ with an intermediate value of 2.8 Mbp blurred the difference between *Aquirufa* species of the *A. antheringensis* and the *A. nivalisilvae* branch concerning the genome sizes (Fig. 3), in contrast, the G + C values of the new strains were between 41.4 and 42.3% and therefore in the range of the values of the other type strains of the *A. antheringensis* branch.

The annotations of the IMG/MER system revealed for all new strains, as usual for *Aquirufa* strains, genes predicted for encoding enzymes of the whole citrate cycle and glycolysis, for syntheses of the carotenoids zeta-carotene, lycopene, beta-carotene, and astaxanthin, as well as several genes for gliding motility and an additional cbb3-type cytochrome c oxidase. The draft genomes of strains KTFRIE-69F^T^ and OSTEICH-129V^T^ comprised genes of the light-harvesting rhodopsin system including besides bacteriorhodopsin a beta-carotene 15,15′-dioxygenase, as it was also found in the type strains of the three so far described species of the *A. antheringensis* branch. The draft genome of strain PLAD-142S6K^T^ included two extra operons relevant to the uptake and use of nitrogen. One operon, which was also found in the genomes of the type strains of *A. antheringensis* and *A. regiilacus*, contained genes predicted for the assimilatory usage of nitrate and nitrite, for example, genes for nitrate reductase, nitrite reductase (nitrite reductase small subunit and nitrite reductase large subunit), a nitrate/nitrite MFS transporter and rubredoxin. In addition, an operon encoding genes predicted for the reduction of nitrous oxide to molecular nitrogen (nitrous-oxide reductase, cytochrome c551/c552, nitrous oxidase accessory protein, copper chaperone NosL) was only found in the draft genome of PLAD-142S6K^T^. The draft genome of strain HETE-83D^T^ encoded a gene annotated as endoglucanase, which can be putatively used for the degradation of cellulose as it was found in the genomes of the type strains of *A. regiilacus* and *A. lenticrescens*.

### Evidence for four new species

The phylogenetic reconstructions based on 16S rRNA gene sequences and amino acid sequences of 501 core genes showed that strains HETE-83D^T^, KTFRIE-69F^T^, OSTEICH-129V^T^, and PLAD-142S6K^T^ belonged to the *A. antheringensis* branch of the genus *Aquirufa*. Calculated gANI values with all possible pairings including all type strains of the branch revealed values between 76.9% and 88.6% for strain HETE-83D^T^, 76.9% and 90.9% for KTFRIE-69F^T^, 76.7% and 79.3% for OSTEICH-129V^T^ and 76.9% and 83.7% for PLAD-142S6K^T^, respectively. All values were thus under the threshold of 95% used for species demarcation of prokaryotes (Konstantinidis et al. 2006; Jain et al. 2018). In addition, we calculated dDDH values with the same set of genomes and obtained values between 19.0 and 42.8%, which were also under the established threshold of 70% (Chun et al. 2018). According to Riesco et al. (Riesco and Trujillo 2024) a genome-based phylogenetic tree should also be involved in defining new species. The phylogenetic reconstructions of Fig. 3 confirmed the finding of the calculated overall genome-related indices, that the four new strains represent four new species. All of them were placed on branches with branch lengths, which means phylogenetic distances, comparable to other *Aquirufa* species or species of the genus *Flectobacillus*. We propose the name *Aquirufa esocilacus* sp. nov. for strain HETE-83D^T^, *Aquirufa originis* sp. nov. for strain KTFRIE-69F^T^, *Aquirufa avitistagni* sp. nov. for strain OSTEICH-129V^T^ and *Aquirufa echingensis* sp. nov. for strain PLAD-142S6K^T^.

### Distribution and ecology of the new species

Isolates representing the four new species were obtained each from only one of the 112 water samples of this study (Table S2). This finding was unexpected as we obtained in this study cultures from nearly all described *Aquirufa* species with type strains isolated from fresh waters in Austria. Since all new strains grow well under the used laboratory conditions, this could mean that the strains represent rare species with special requirements regarding environmental conditions of habitat type or that they occur only in low abundances. Since BLAST searches with 16S rRNA gene sequences are not appropriate for getting information about the distribution of a particular species (see above), analyzing publicly available metagenome sequence data by mapping could be a way to get this information. Thereby it should be borne in mind that these sequence data were generated without PCR steps, so species that occur in low abundances cannot be detected. We calculated the percentages of reads mapped on our genomes, to estimate the relative abundances of the new species, so the portion they had on the bacterial community in the samples. Table S6 shows all data of the considered metagenomes with detection for at least one of the new species. The new species *A. esocilacus* could only be detected in water samples of a small pond in Uppsala, Sweden. The new species *A. originis* appeared in the same habitat and was also detected in the Jinsha River, China (Liu et al. 2020). Genomic DNA of the new species *A. echingensis* appeared in water samples of the Torrens River, Australia and over a length of more than 3000 km at different sites of the Yangtze River, China (Liu et al. 2020). The new species *A. avitistagni* was detected in water samples from various sites in Asia, including Lake Lugu, China (Shen et al. 2019), River Geum, Republik Korea (Shim et al. 2018) and different sites along the Tongtian River (Zhao et al. 2019), Jinsha River and Yangtze River (Liu et al. 2020), China. While Uppsala (Sweden) like Austria is located in the temperate climate zone, all other sites are in the subtropical climate zone. Interestingly, the new species *A. esocilacus* and *A. originis* could be detected only in the temperate climate zone or at higher altitudes (1817 m) in the subtropical climate zone. That could be a hint that these species prefer moderate temperatures. The portions of the *Aquirufa* species on the bacterial communities seemed to be small in most cases (0.003–0.105% mapped reads, Table S6). For *A. echingensis* three higher values (0.207, 0.302, 0.845%) were detected for samples of the Yangtze River, China, nevertheless, these values suggested that the species does not represent a dominant taxon in the samples.

While all new strains were isolated from small standing or running water bodies the data above showed that the species *A. echingensis* and *A. avitistagni* colonize large water bodies as well. This could be evidence of a pelagic lifestyle, which corresponds with the relatively low genome sizes of the new strains (Chiriac et al. 2023). The draft genomes of strains KTFRIE-69F^T^ and OSTEICH-129V^T^ contained genes putatively encoding a light-harvesting rhodopsin system, which could be useful in upper water layers, where enough light is available. The draft genome of PLAD-142S6K^T^ contained an operon with genes predicted for the reduction of nitrous oxide to molecular nitrogen, which is the last step of microbial nitrate respiration. Since nitrous oxide occurs under low oxygen or anoxic conditions (Wang et al. 2021), probably the strain may tolerate conditions at the aerobe/anaerobe interface which corresponds with the observed weak growth under anaerobic laboratory conditions.

### Description of *Aquirufa esocilacus* sp. nov.

*Aquirufa esocilacus* (e.so.ci.la’cus. L. masc. n. *esox*, a kind of fish; L. masc. n. *lacus*, lake; N.L. gen. n. *esocilacus*, of a lake named after *Esox* fish).

Cultures of the type strain grown under aerobic conditions (21 °C) on NSY medium have the following characteristics: cells form rods with an approximate size of 1.1 × 0.4 µm. Liquid cultures have a red–orange coloring, and colonies on agar plates are red-pigmented. Cultures show motility on soft agar plates and grow at 5–31 °C and do not grow above 0% (w/v) NaCl or under anaerobic conditions. Major fatty acids are iso-C_15:0_, anteiso-C_15:0_, C_16:1_ω5c and C_16:1_ω7c. Polar lipids are phosphatidylethanolamine besides unidentified phospholipids, aminophospholipids, glycolipids and further polar lipids. Menaquinone-7 occurs as major respiratory quinone, menaquinone-6 in small amounts and traces of menaquinone-8 may occur. The genomic DNA of the type strain has 2.6 Mbp and a G + C content of 41.8%.

The type strain is HETE-83D^T^ (= DSM 118087^ T^ = JCM 37094^ T^), isolated from a small pond in Obertrum (Salzburg, Austria).

Sequence accession numbers: 16S rRNA gene PQ394670.2, whole genome JBBKXX000000000.

### Description of *Aquirufa originis* sp. nov.

*Aquirufa originis* (o.ri’gi.nis. L. gen. n. *originis*, of the origin, named after the school HBLA Ursprung (origin), which was involved in the isolation of the strain).

Cultures of the type strain grown under aerobic conditions (21 °C) on NSY medium have the following characteristics: Cells form rods with an approximate size of 1.1 × 0.4 µm. Liquid cultures have a red–orange coloring, colonies on agar plates are red-pigmented. Cultures show motility on soft agar plates and grow at 5–31 °C and do not grow above 0% (w/v) NaCl or under anaerobic conditions. Major fatty acids are iso-C_15:0_, C_16:1_ω7c and anteiso-C_15:0_. Polar lipids are phosphatidylethanolamine as well as unidentified phospholipids, aminophospholipids, unidentified glycolipids and further polar lipids. Menaquinone-7 occurs as major respiratory quinone, menaquinone-6 in small amounts and traces of menaquinone-8 may occur. The genomic DNA of the type strain has 2.5 Mbp and a G + C content of 42.3%.

The type strain is KTFRIE-69F^T^ (= DSM 117798^T^ = JCM 37095^T^), isolated from a small artificial pond in Gloednitz (Carinthia, Austria).

Sequence accession numbers: 16S rRNA gene PQ394674.2, whole genome JBBKXY000000000.

### Description of *Aquirufa avitistagni* sp. nov.

*Aquirufa avitistagni* (a.vi.ti.stag'ni. L. masc. adj. *avitus*, belonging to a grandparent, ancestral; L. neut. n. stagnum, pond; N.L. gen. n. *avitistagni*, of a pond belonging to the grandmother of the person who took the water sample).

Cultures of the type strain grown under aerobic conditions (21 °C) on NSY medium have the following characteristics: Cells form rods with an approximate size of 1.4 × 0.4 µm. Liquid cultures form red–orange suspensions with tiny orange globules, and colonies on agar plates are red-pigmented. Cultures show no motility on soft agar plates and grow at 5–30 °C and in 0–0.1% (w/v) NaCl and do not grow under anaerobic conditions. Major fatty acids are iso-C_15:0_, C_16:1_ω7c and anteiso-C_15:0_. Polar lipids are phosphatidylethanolamine as well as unidentified aminophospholipids, glycolipids and further polar lipids. Menaquinone-7 occurs as major respiratory quinone, menaquinone-6 in small amounts and traces of menaquinone-8 may occur. The genomic DNA of the type strain has 2.6 Mbp and a G + C content of 41.8%.

The type strain is OSTEICH-129V^T^ (= DSM 118088^ T^ = JCM 37100^ T^), isolated from a garden pond in Rutzenmoos (Upper Austria, Austria).

Sequence accession numbers: 16S rRNA gene PQ394675.2, whole genome JBBKXZ000000000.

### Description of *Aquirufa echingensis* sp. nov.

*Aquirufa echingensis* (ech.ing.en'sis. N.L. fem. adj. *echinensis*, pertaining to Eching, near Salzburg in Austria).

Cultures of the type strain grown under aerobic conditions (21 °C) on NSY medium have the following characteristics: Cells form rods with an approximate size of 1.6 × 0.5 µm. Liquid cultures have a red–orange coloring, and colonies on agar plates are red-pigmented. Cultures show motility on soft agar plates, grow at 5–32 °C, in 0–0.2% (w/v) NaCl and grow weakly under anaerobic conditions on NSY agar plates. Major fatty acids are iso-C_15:0_, anteiso-C_15:0_ and C_16:1_ω7c. Polar lipids are phosphatidylethanolamine besides unidentified aminophospholipids, glycolipids and further polar lipids. Menaquinone-7 occurs as major respiratory quinone, menaquinone-6 in small amounts and traces of menaquinone-8 may occur. The genomic DNA of the type strain has 2.8 Mbp and a G + C content of 41.4%.

The type strain is PLAD-142S6K^T^ (= DSM 117799^T^ = JCM 37096^T^), isolated from a creek in St. Georgen (Salzburg, Austria).

Sequence accession numbers: 16S rRNA gene PQ394676.2, whole genome JBBKYA000000000.

## Supplementary Information

Below is the link to the electronic supplementary material.

Supplementary file1 (PDF 873 KB)

## Data Availability

The Whole Genome Shotgun project has been deposited at DDBJ/ENA/GenBank under the accession JBBKXX000000000 for strain HETE-83DT, JBBKXY000000000 for strain KTFRIE-69FT, JBBKXZ000000000 for strain OSTEICH-129VT, and JBBKYA000000000 for strain PLAD-142S6KT. These are the versions described in this paper. The accession of the 16S rRNA gene sequence deposited at DDBJ/ENA/GenBank of strain HETE-83DT are PQ394670.2, of strain KTFRIE-69FT PQ394674.2, of strain OSTEICH-129VT PQ394675.2, and of strain PLAD-142S6KT PQ394676.2.

## Abbreviations

*A.*: *Aquirufa*
gANI: Whole-genome average nucleotide identity
NSY medium: Nutrient broth-soytone-yeast extract medium
*gyrB*: B subunit of the DNA gyrase
GC/MS: Gas chromatography/mass spectrometry
IMG/MER: Integrated Microbial Genomes & Microbiomes Expert Review
