# Supplementary material for: Aquirufa esocilacus sp. nov., Aquirufa originis sp. nov., Aquirufa avitistagni, and Aquirufa echingensis sp. nov. discovered in small freshwater habitats in Austria during a citizen science project
Source: Arch Microbiol. 2025 Feb 25;207(4):71. doi: 10.1007/s00203-025-04275-6 (PMC11861422; doi:10.1007/s00203-025-04275-6)
Supplement: Supplementary file 1 — Supplementary file1 (PDF 873 KB) [file 203_2025_4275_MOESM1_ESM.pdf]

Supplementary Information

Archives of Microbiology

Aquirufa esocilacus sp. nov., *Aquirufa originis* sp. nov., *Aquirufa avitistagni*, and *Aquirufa echingensis* sp. nov. discovered in small freshwater habitats in Austria during a citizen science project

Alexandra Pitt, Stefan Lienbacher, Johanna Schmidt, Meina Neumann-Schaal, Jacqueline Wolf, Aharon Oren, Sophia Reichl and Martin W. Hahn

Correspondence: Alexandra Pitt, alexandra.pitt@uibk.ac.at

Table S1 Accession numbers and IMG/MER identity numbers of the genomes used for comparison and the RAxMl phylogenetic tree of Fig. 3; n. a., not available

Species and strain	NCBI accession number	IMG/MER ID
Aquirufa regiilacus LEOWEIH-7C^{T}	JAVNWW000000000	8023692520
Aquirufa echingensis PLAD-142S6K^{T}	JBBKYA000000000	8069800526
Aquirufa avitistagni OSTEICH-129V^{T}	JBBKXZ000000000	8076109451
Aquirufa lenticrescens 9H-EGSE^{T}	CP049834	2857132225
Aquirufa esocilacus KTFRIE-69F^{T}	JBBKXY000000000	8069795670
Aquirufa antheringensis 30S-ANTBAC^{T}	SEWZ00000000	2816332120
Aquirufa originis HETE-83D^{T}	JBBKXX000000000	8069793293
Aquirufa nivalisilvae 59G-WUEMPEL^{T}	SEWX00000000	2816332125
Aquirufa aurantiipilula 15D-MOB^{T}	JAANOG000000000	2857134496
Aquirufa ecclesiirivi 50A-KIRBA^{T}	JAANOP000000000	2828879446
Aquirufa beregesia 50C-KIRBA^{T}	SEWW00000000	2816332124
Aquirufa rosea CAR-16^{T}	SDHY00000000	2844599478
Sandaracinomonas limnophila LMG 29732^{T}	SACY00000000	2829520394
Arcicella rigui KCTC 23307^{T}	JAYFUM000000000	n. a.
Arcicella aquatica LMG 21963^{T}	JAYFUL000000000	n. a.
Arcicella rosea DSM 21163^{T}	n. a.	2928561906
Arcicella aurantiaca LMG 25207^{T}	QGGO00000000	2595698202
Flectobacillus roseus LMG 24501^{T}	JASHIG000000000	8055515397
Flectobacillus rivi KCTC 92562^{T}	JASHIE000000000	n. a.
Flectobacillus longus KCTC 92561^{T}	JASHIC000000000	8085389167
Flectobacillus major DSM 103^{T}	ATXY00000000	2509601041
Pseudarcicella hirudinis LMG 26720^{T}	FOXH00000000	2634166314

Table S2 Data of the sampled habitats from Fig. 1 and taxonomic assignment of the obtained cultures. Last column: 1, *A. antheringensis*; 2, *A. regiilacus*; **3**, *A. originis*; **4**, *A. esocilacus*; **5**, *A. avitistagni*; **6**, *A. echingensis*; 7, *A. nivalisilvae*; 8, *A. beregesia*; 9, *A. ecclesiirivi*; 10, *A. aurantiipilula*, 11, *Aquirufa* sp.

No.	Habitat ID	Name of habitat	Type of habitat	Latitude (N)	Longitude (E)	Date of sampling	pH	Con-ductivity (µS/cm)	Culture/s obtained	Belonging to species
1	LEIBA	Leitenbach	creek	47.780278	13.224444	12/11/2022	6.5	408	no	-
2	AUSEE	Ausee	lake	47.920803	12.958752	13/11/2022	6.6	374	no	-
3	LEPPI	Leopoldskroner Weiher	lake	47.784450	13.038460	14/11/2022	6.9	274	yes	2
4	MOBA	Morzger Bach	creek	47.778600	13.058000	15/11/2022	7.2	872	no	-
5	MRZGBC	Morzger Bach	creek	47.778600	13.058000	15/11/2022	7.2	603	yes	9
6	LEOWEIH	Leopoldskroner Weiher	lake	47.783260	13.041110	16/11/2022	7.0	399	yes	2
7	FUSSEE	Fuschlsee	lake	47.792860	13.297800	13/11/2022	7.1	630	yes	7
8	BACHDPS	Bach in Aigen (Salzburg)	creek	47,798212	13.081885	15/11/2022	6.9	398	no	-

9	SALZBACH	Salzquellenbach	creek	47.704081	13.066418	15/11/2022	6.9	581	no	-
10	WALLSEE	Wallersee	lake	47.915911	13.177321	15/11/2022	6.8	228	yes	1
11	GLASBACH	Glasbach	creek	47.797808	13.064742	13/11/2022	7.2	678	yes	9
12	KUHSEE	Kuchler See	lake	47.622013	13.141347	15/11/2022	6.5	278	no	-
13	KSACHE	Königsseeache	stream	47.729917	13.070917	13/11/2022	6.9	336	no	-
14	GLAN	Glanbach	stream	47.791890	13.020000	13/11/2022	6.7	385	no	-
15	AUTBSEE	Autobahnsee	lake	47.778370	12.986280	15/11/2022	6.9	418	no	-
16	GLASBA	Glasbach	creek	47.767222	13.079361	16/11/2022	6.2	302	no	-
17	WAEICH	Waldteich	pond	47.777804	12.946487	15/11/2022	6.9	472	yes	10
18	KEE	Kuchler See	lake	47.622013	13.141347	15/11/2022	6.5	371	no	-
19	LKW	Leopoldskroner Weiher	lake	47.789210	13.040846	13/11/2022	6.9	297	no	-
20	TRAUN	Traun	river	48.023167	13.808000	21/11/2022	6.3	261	no	-
21	VKA	Vöckla	river	48.010356	13.652875	22/11/2022	NA	256	no	-
22	STADI	Rainwater	rain	48.007745	13.653751	22/11/2022	6.1	91	no	-
23	VÖLCKA	Vöckla	river	48.004423	13.670058	22/11/2022	6.6	361	no	-
24	ROETBACH	Rötelbach	creek	48.087897	13.665738	22/11/2022	6.6	433	no	-
25	HOERACH	Höribach	creek	47.842161	13.337258	22/11/2022	6.9	163	no	-
26	AGELIX	Ager	river	47.993944	13.652778	21/11/2022	6.7	377	no	-
27	AGERI	Ager	river	47.994724	13.657023	22/11/2022	6.6	426	no	-
28	BGSTV	School pond	pond	48.008005	13.668461	23/11/2022	6.8	321	no	-
29	SIMRING	Source Redelbach	source	48.128287	13.624384	21/11/2022	6.5	452	no	-
30	BHBGOP	Bach bei Gopprechting	creek	48.006088	13.511044	20/11/2022	7.8	328	yes	8. 9
31	GTM	Garden pond Miglberg	pond	47.902416	13.603627	20/11/2022	7.5	322	no	-
32	GARBAGER	Garden pond	pond	48.001067	13.732363	22/11/2022	7.0	236	yes	7
33	WATS	Forest pond Schönberg	pond	47.978611	13.658889	20/11/2022	5.5	114	yes	7
34	AGERII	Ager	river	47.994361	13.656361	21/11/2022	6.5	469	no	-
35	AUBACH	Aubach	creek	47.978480	13.698385	10/11/2022	6.6	126	no	-
36	BAPO	Bach an Postleiden	creek	47.973996	13.712443	22/11/2022	6.6	258	no	-
37	POOLCB	Garden pool	artificial pool	47.978333	13.698333	09/11/2022	5.5	8270	no	-
38	POHNTON NE	Rain barrel in Pohnedt	rain barrel	48.039146	13.616410	22/11/2022	7.5	1041	no	-
39	DÜMPEL	Forest pond	pond	48.043028	13.609194	19/11/2022	7.7	444	no	-
40	HIBACH	Hinterbach	creek	48.057750	13.784074	22/11/2022	7.2	550	no	-
41	BABACH	Bach in Bach	creek	48.055212	13.703071	12/11/2022	7.1	550	yes	9
42	AGERTUE	Ager-pond	pond	47.997222	13.649444	21/11/2022	6.6	393	yes	1
43	AGERIII	Ager	river	47.993731	13.712366	12/11/2022	7.5	639	no	-
44	AGERIV	Ager	river	47.995371	13.709739	12/11/2022	7.5	548	no	-

45	DARE	Rain barrel	rain barrel	48.009244	13.719426	22/11/2022	5.5	50	yes	7
46	HEBA	Heißingbach	creek	47.767841	13.105447	07/04/2023	6.6	403	yes	9
47	KIBA	Kirchstättbach	creek	47.938744	13.070284	11/04/2023	6.3	326	no	-
48	HINT	Hintersee	lake	47.749217	13.251770	11/04/2023	6.5	423	no	-
49	URTEICH	Pond in Ursprung	pond	47.880451	13.063230	11/04/2023	6.3	336	no	-
50	FEUEUG	Wet medow in Eugendorf	wet medow	47.873056	13.119444	10/04/2023	6.7	231	yes	1
51	BAGRA	Creek in Grabensee	creek	48.000035	13.092802	11/04/2023	6.9	591	yes	7, 9
52	KLABACH	Klammbach	stream	47.363847	11.116872	11/04/2023	6.9	502	no	-
53	RWSWB	Reservoir in Weichenlang	water reservoir	47.870000	13.062139	11/04/2023	6.3	69	no	-
**54**	KTFRIE	Pond at Friesserhof	pond	46.894944	14.123163	11/04/2023	5.5	64	yes	3
55	REITQU	Source Vorderreit	source	47.702782	13.197628	NA	6.8	306	no	-
56	WALLSEE	Wallersee	lake	47.926389	13.208889	11/04/2023	6.6	334	yes	1
57	TRAUNSE	Traunsee	lake	47.810278	13.790833	11/04/2023	6.9	358	no	-
58	IBSEE	Heratinger See	lake	48.072336	12.952950	10/04/2023	6.9	381	no	-
59	AUSIEZ	Autobahnsee	lake	47.807263	12.973135	05/04/2023	6.3	131	no	-
60	HASAL	Salzach	river	47.685541	13.094385	04/04/2023	6.2	255	no	-
61	ROBBACH	Creek in Robingstraße	creek	47.815872	13.061949	10/04/2023	7.2	601	yes	1, 7
62	NATPOOL	Swimming pond	pond	48.406851	14.470661	11/04/2023	6.3	193	no	-
63	TUEMPMO	Pond	pond	47.691179	13.133468	10/04/2023	6.7	343	no	-
**64**	HETE	Hechtteich at Gauesed	pond	47.914312	13.015339	11/04/2023	7.0	90	yes	4, 9
65	GARMA	Garden pond in Ochsenharing	pond	47.962790	13.100011	10/04/2023	6.3	271	yes	1
66	LEICHEB	Leichertinger Bach	creek	47.858534	13.064505	11/04/2023	6.1	252	no	-
67	WTV	Rain barrel	rain barrel	47.967959	13.099646	11/04/2023	5.5	57	no	-
68	WIEBACH	Wiesenbach	creek	47.912017	13.214282	11/04/2023	6.7	620	no	-
69	RETO	Rain barrel	rain barrel	48.019376	13.094285	10/04/2023	5.5	373	no	-
70	QUEOS	Source in Rutzenmoos	source	47.94554	13.72464	02/05/2023	5.2	236	no	-
71	VOEKFU	Vöckla	river	48.006806	13.660111	08/05/2023	6.9	325	yes	1, 2
72	GRIRUBA	Griebach	creek	47.969220	13.707350	29/04/2023	7.2	432	yes	7, 10
73	ZURAUKO	Hüttwinklache	stream	47.075579	12.980358	04/05/2023	6.3	49	no	-
74	LITZATS	Attersee	lake	47.926243	13.548776	07/05/2023	6.9	222	no	-
75	WANK-RACH	Aurach	creek	48.000076	13.734035	08/05/2023	7.2	354	yes	9
76	DURAGER	Dürre Ager	river	47.997740	13.609002	08/05/2023	6.6	519	yes	1
77	ROETBACH	Rötelbach	creek	48.095680	13.661130	06/05/2023	7.2	487	yes	1

78	ATTSEE	Attersee	lake	47.948752	13.594745	08/05/2023	6.2	308	no	-
79	FRTEICH	Pond in Friesam	pond	48.117889	13.698373	NA	6.8	398	yes	1
80	HOEBACH	Höribach	creek	48.015890	13.718020	08/05/2023	6.8	233	no	-
81	AGR	Ager	river	48.058016	13.796355	07/05/2023	7.2	459	yes	1
82	VOEKLAB	Vöckla	river	48.007510	13.660530	NA	7.5	318	yes	1
83	WAICH	Wankhamer pond	pond	47.998717	13.732549	08/05/2023	6.8	46	no	-
84	WAIG	Creek in Gsperr	creek	47.913890	13.817160	08/05/2023	6.8	597	no	-
85	DUEGER	Dürre Ager	river	47.994167	13.577500	08/05/2023	7.8	85	yes	1
86	LACHER	Laudachsee Moor	bog pond	47.876150	13.853070	01/05/2023	4.9	19	no	-
87	VOEWA	Vöckla	river	48.001000	13.650000	05/06/2023	5.5	442	yes	10
88	REGETON	Rain barrel	rain barrel	48.073395	13.489823	05/06/2023	9.4	409	no	-
89	GAMSEE	Garden pond in Gampern	pond	47.988243	13.554729	05/06/2023	5.7	108	no	-
90	VCK	Vöckla	river	48.010935	13.657192	05/06/2023	6.7	92	yes	1
91	FRUEMPEL	Forest pond	pond	48.069164	13.515403	04/06/2023	6.5	79	no	-
92	ATSEE	Attersee	lake	47.937501	13.593056	05/06/2023	7.2	108	no	-
93	WAALT	Brunngraben	ditch	48.015186	13.624639	05/06/2023	7.2	320	no	-
**94**	OSTEICH	Garden pond	pond	47.968330	13.699821	05/06/2023	6.2	42	yes	5, 7, 11
95	SCHWONA	Hinterbach	creek	48.058765	13.782329	05/06/2023	8.2	534	no	-
96	STNV	Swimming pond	pond	48.010942	13.657880	06/06/2023	6.9	190	no	-
97	SCO8	Rain puddle	rain puddle	48.056389	13.772148	06/06/2023	6.1	192	no	-
98	GZH	Water well	well	47.936111	13.590000	05/06/2023	6.6	48	yes	1
99	FREDL	Redl	creek	48.066362	13.490258	04/06/2023	6.4	257	no	-
100	TEIWEDO	Pond in Webersdorf	pond	48.101528	12.952417	18/06/2023	6.5	253	yes	1
**101**	PLAD	Pladenbach	creek	47.971592	12.889386	12/06/2023	6.8	451	yes	1, 6, 9
102	IBMMO	Ibmer-Moor	bog pond	48.053609	12.959757	18/06/2023	5.2	62	no	-
103	SALCH	Salzach	river	47.943303	12.939030	19/06/2023	NA	194	yes	1
104	HATUE	Handenberg pond	pond	48.136593	13.001099	18/06/2023	8.1	288	yes	1, 9
105	BACHBUER	Creek at Bürmoos	creek	47,983329	12.913086	17/06/2023	6.9-7.2	497	yes	1
106	SAAGRA	Saaggraben	creek	48.067542	12.981010	18/06/2023	6.9	311	yes	1, 7, 9, 10
107	MUNTEICH	Garden pond in Muntigl	garden pond	47.852320	13.015210	NA	6.7	177	no	-
108	ECHBACH	Creek in Eching	creek	47.975044	12.884126	18/06/2023	4.5	377	yes	1, 9
109	RETEI	Garden pond	garden pond	47.995328	12.959356	19/06/2023	7.2	230	no	-
110	MO-P	Mondsee	lake	47.807666	13.387441	16/06/2023	7.9	335	yes	1
111	GUE 29	Gosausee	lake	47.532101	13.498617	18/06/2023	6.6	-	no	-
112	GUE 30	Hallstättersee	lake	47.594941	13.651762	18/06/2023	6.9	-	yes	1, 2

Table S3 Fatty acid composition of strains HETE-83D^T, KTFRIE-69F^T, OSTEICH-129V^T, and PLAD-142S6K^T. Only fatty acids with values ≥ 1 % for at least one strain were listed. Values of > 10 % were marked with bold letters (major fatty acids).

	HETE-83D^T	KTFRIE-69F^T	OSTEICH-129V^T	PLAD-142S6K^T
$C_{14:0}$	1.5	1.3	4.1	0.8
$C_{14:1}\omega3c$	1.0	0.3	1.6	0.6
$C_{15:0}$	0.2	0.4	-	2.3
$C_{15:1}\omega4c$ (Unknown 14.959)	4.5	1.8	0.2	6.1
$C_{15:1}\omega6c$	1.3	1.1	0.2	4.9
$C_{16:0}$	0.2	0.2	1.0	0.1
$C_{16:0}$3-OH	0.4	-	1.3	0.7
$C_{16:1}\omega5c$	**11.1**	7.2	7.9	5.3
$C_{16:1}\omega7c$	**10.6**	**11.7**	**18.4**	**12.8**
$C_{17:1}\omega5c$	1.5	-	-	-
$C_{17:1}\omega6c$	0.6	0.7	-	1.9
iso-$C_{11:0}$	2.3	2.2	1.3	2.6
iso-$C_{15:0}$	**40.2**	**45.2**	**34.3**	**28.8**
anteiso-$C_{15:0}$	**14.4**	**10.2**	**14.0**	**13.0**
anteiso-$C_{15:0}$3-OH	-	-	1.6	-
iso-$C_{15:1}\omega5c$	0.5	1.0	0.8	0.8
iso-$C_{17:1}\omega5c$	-	2.3	0.8	1.1
iso-$C_{17:1}\omega7c$	-	1.4	0.4	0.7
iso-$C_{15:0}$3-OH	3.6	7.2	6.7	7.1
iso-$C_{17:0}$3-OH	0.7	1.9	1.3	0.9

Table S4 16S rRNA gene sequence similarities of strains HETE-83D^T, KTFRIE-69F^T, OSTEICH-129V^T, and PLAD-142S6K^T and the nearest related type strains. For calculation the whole 16S rRNA gene (1506 bp) was used, the calculations were performed in the IMG/MER system (Chen et al. 2019).

	HETE-83D^T	KTFRIE-69F^T	OSTEICH-129V^T	PLAD-142S6K^T
Aquirufa antheringensis	100	100	99.3	99.4
Aquirufa lenticrescens	99.9	99.9	99.3	99.4
Aquirufa regiilacus	99.4	99.4	99.9	100
HETE-83D^T	-	100	99.4	99.5
KTFRIE-69F^T	100	-	99.4	99.5
OSTEICH-129V^T	99.4	99.4	-	99.9
PLAD-142S6K^T	99.5	99.5	99.9	-

Table S5 Features of the draft genomes of the new strains.

	HETE-83D^T	KTFRIE-69F^T	OSTEICH-129V^T	PLAD-142S6K^T
Genome size (Mbp)	2.6	2.5	2.6	2.8
G+C content (mol%)	41.8	42.3	41.8	41.4
Number of contigs	11	11	14	14
Nucleotide coverage	727 x	1227.9 x	837.9 x	786.2 x
L50 value	2	2	1	3
Completeness (%)	94.2	94.2	94.8	96.0
Contamination (%)	0.0	0.1	0.0	0.0

Table S6 Detections of the four new species in publicly available metagenome datasets.

Habitat	Latitude Longitude ASL	Sampling Date	pH	Cond. (µS/cm)	T (°C)	Accession number run	Size of meta-genome (Gbp)	Reference	Detection of species	Reads mapped (%)
Pond in Uppsala, Sweden	59.852955 N<br>17.640972 E<br>15 m	July 2021	n.d.	n.d.	19.6	SRR26965952<br>SRR26965953	63.6<br>51.6	No reference	*A. originis*<br>*A. esocilacus*	0.024/0.044<br>0.033/0.055
Lake Lugu, China	27.71-27.73 N<br>100.76-100.8 E<br>2685 m	August 2017	8.8	210	20.9	SRR9302957	41.4	(Shen et al. 2019)	*A. avitistagni*	0.025
River Geum, Republik Korea	36.461306 N<br>127.095889 E<br>8 m	2016	8.0	350	n.d.	SRR12487025	16.6	(Shim et al. 2018)	*A. avitistagni*	0.048
Torrens River, Australia	34.917864 S<br>138.588777 E<br>21 m	January 2021	7.6	1830	23.4	SRR22711026	26.4	No reference	*A. avitistagni*	0.050
Torrens River, Australia	34.917864 S<br>138.588777 E<br>21 m	January 2021	7.8	1760	26.6	SRR22711027	26.0	No reference	*A. avitistagni*	0.105
Torrens River, Australia	34.917864 S<br>138.588777 E<br>21 m	February 2021	6.9	367	20.5	SRR22711023	21.4	No reference	*A. echingensis*	0.069
Tongtian River, China	33.01 N<br>97.24 E<br>3529 m	July 2017	8.5	1154	n.d.	SRR9924779	15.2	(Zhao et al. 2019)	*A. avitistagni*	0.031
Jinsha River, China	26.88 N<br>99.98 E<br>1817 m	October 2014	8.2	488	n.d.	SRR9924778	16.8	(Liu et al. 2020) Environmental data: (Yang et al. 2020)	*A. avitistagni*<br>*A. originis*	0.048<br>0.046
Jinsha River, China	26.569125 N<br>101.701627 E<br>988 m	October 2014	8.2	488	n.d	SRR9924781	17.4	(Liu et al. 2020) Environmental data: (Yang et al. 2020)	*A. echingensis*<br>*A. avitistagni*	0.006<br>0.044
Yangtze River, China	28.77 N<br>104.65 E<br>259 m	October 2014	8.0	405	n.d.	SRR9924785	14.9	(Liu et al. 2020) Environmental data: (Lu et al. 2023)	*A. echingensis*	0.017
Yangtze River, China	28.897665 N<br>105.551587 E<br>217 m	October 2014	7.1	410	n.d	SRR9924784	14.3	(Liu et al. 2020) Environmental data: (Lu et al. 2023)	*A. echingensis*<br>*A. avitistagni*	0.030<br>0.039

Yangtze River, China	29.019678 N<br>105.85225 E<br>191 m	October 2014	7.8	410	n.d.	SRR9924799	16.4	(Liu et al. 2020) Environmental data: (Lu et al. 2023)	*A. echingensis*<br>*A. avitistagni*	0.039<br>0.048
Yangtze River, China	30.886945 N<br>110.905195<br>156 m	October 2014	7.9	390	n.d.	SRR9924803	17.4	(Liu et al. 2020) Environmental data: (Lu et al. 2023)	*A. echingensis*<br>*A. avitistagni*	0.002<br>0.019
Yangtze River, China	29.62 N<br>106.6 E<br>155 m	October 2014	8.0	405	n.d.	SRR9924798	14.3	(Liu et al. 2020) Environmental data: (Lu et al. 2023)	*A. echingensis*<br>*A. avitistagni*	0.042<br>0.083
Yangtze River, China	31.04 N<br>110.4 E<br>63 m	October 2014	7.9	390	n.d.	SRR9924797	19.6	(Liu et al. 2020) Environmental data: (Lu et al. 2023)	*A. echingensis*<br>*A. avitistagni*	0.011<br>0.021
Yangtze River, China	30.69 N<br>111.28 E<br>36 m	October 2014	7.9	400	n.d.	SRR9924791	12.6	(Liu et al. 2020) Environmental data: (Lu et al. 2023)	*A. echingensis*	0.302
Yangtze River, China	30.69 N<br>111.28 E<br>36 m	October 2014	7.9	390	n.d.	SRR9924800	12.4	(Liu et al. 2020) Environmental data: (Lu et al. 2023)	*A. echingensis*	0.207
Yangtze River, China	30.29 N<br>112.26 E<br>28 m	October 2014	7.8	340	n.d.	SRR9924790	15.0	(Liu et al. 2020) Environmental data: (Lu et al. 2023)	*A. echingensis*<br>*A. avitistagni*	0.062<br>0.024
Yangtze River, China	29.45 N<br>113,15 E<br>21 m	October 2014	7.9	300	n.d.	SRR9924755	31.1	(Liu et al. 2020) Environmental data: (Lu et al. 2023)	*A. echingensis*	0.003
Yangtze River, China	30.62 N<br>114.32 E<br>20 m	October 2014	7.8	350	n.d.	SRR9924753	14.1	(Liu et al. 2020) Environmental data: (Lu et al. 2023)	*A. echingensis*	0.845
Yangtze River, China	29.670001 N<br>113.319994 E<br>19 m	October 2014	7.9	300	n.d.	SRR9924756	12.5	(Liu et al. 2020) Environmental data: (Lu et al. 2023)	*A. echingensis*	0.009
Yangtze River, China	31.77 N<br>118.47 E<br>1 m	October 2014	7.9	330	n.d.	SRR9924757	13.4	(Liu et al. 2020) Environmental data: (Lu et al. 2023)	*A. echingensis*	0.003

Fig. S1 Boxplot of gyrB gene sequence similarities between same species and different species of the genus *Aquirufa*. All considered strains are genome sequenced and assigned to species by calculating gANI values with the type strains of the genus. Shown is the minimum, maximum, median, lower quartile and upper quartile. The blue line indicates the proposed demarcation of 92 % used for searching for new species. The figure shows, that *gyrB* gene sequence similarity values around 95 % occur in both groups.

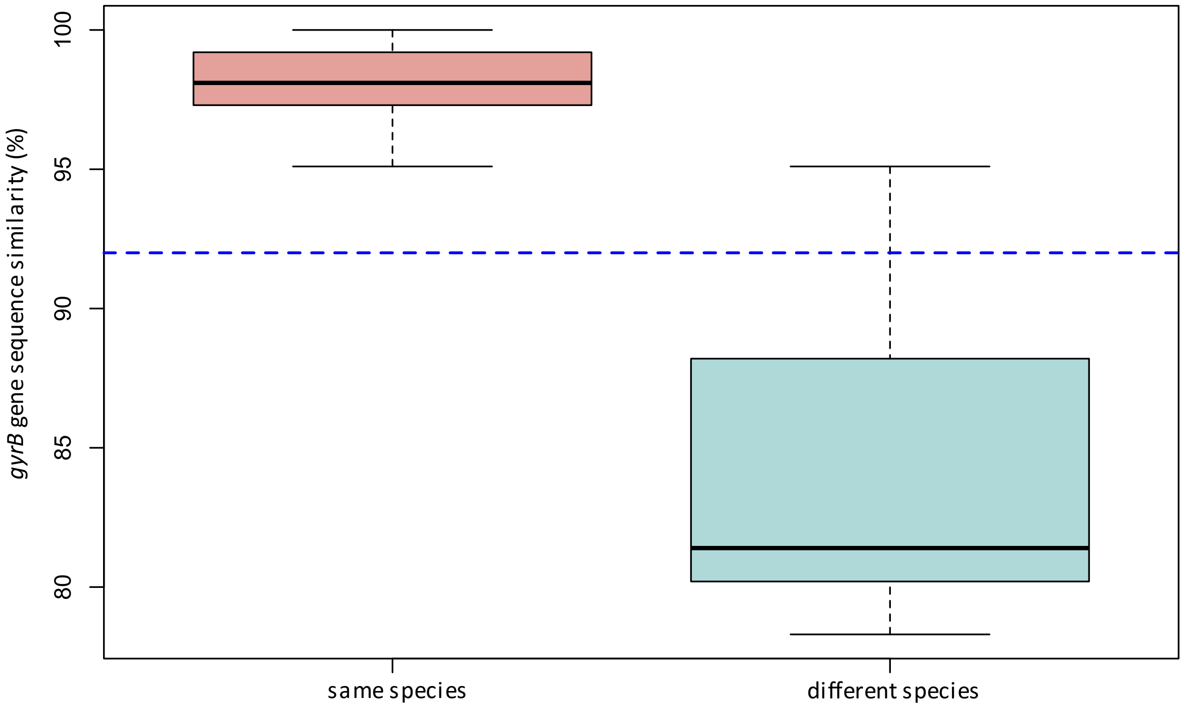

Fig. S2 Mapping of *Polynucleobacter paneuropeus* strains on metagenomes harboring genomes of *Polynucleobacter paneuropaeus* (i.e., mapping results in coverage depth > 100-fold).

The number of mapped metagenomic reads was stepwise reduced and the respective coverage depth and breadth were recorded (see main text).

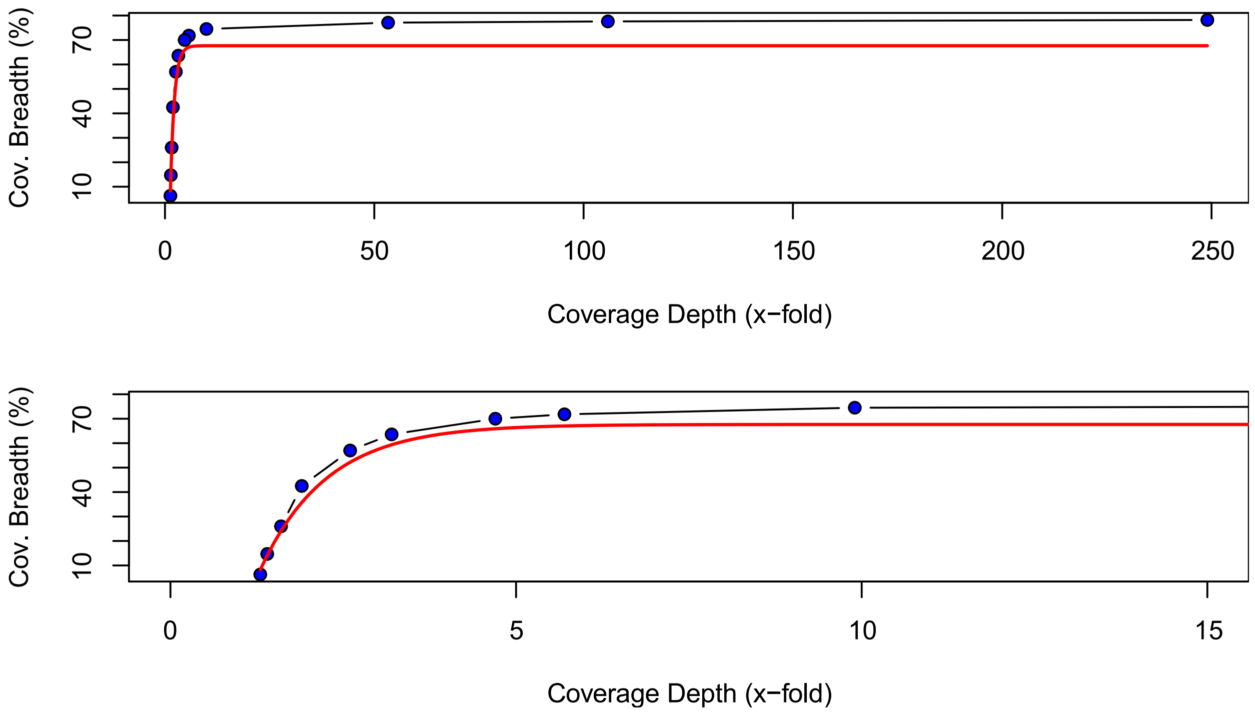

Fig. S3 Polar lipid pattern of strains HETE-83D^{T}, KTFRIE-69F^{T}, OSTEICH-129V^{T}, and PLAD-142S6K^{T}.

For each strain: first row: left side, visualization of total lipids with dodecamolybdophosphoric acid; right side: visualization of glycolipids with α-naphthol Second row: left side, visualization of phospholipids with molybdenum blue; right side, visualization of aminolipids with ninhydrin.

F24-236, HETE-83D^{T}; F24-235, KTFRIE-69F^{T}; F24-238, OSTEICH-129VT; F24-237, PLAD-142S6K^{T}.

PE, phosphatidylethanolamine; APL, aminophospholipid; GL, glycolipid; PL, phospholipid; L, lipid

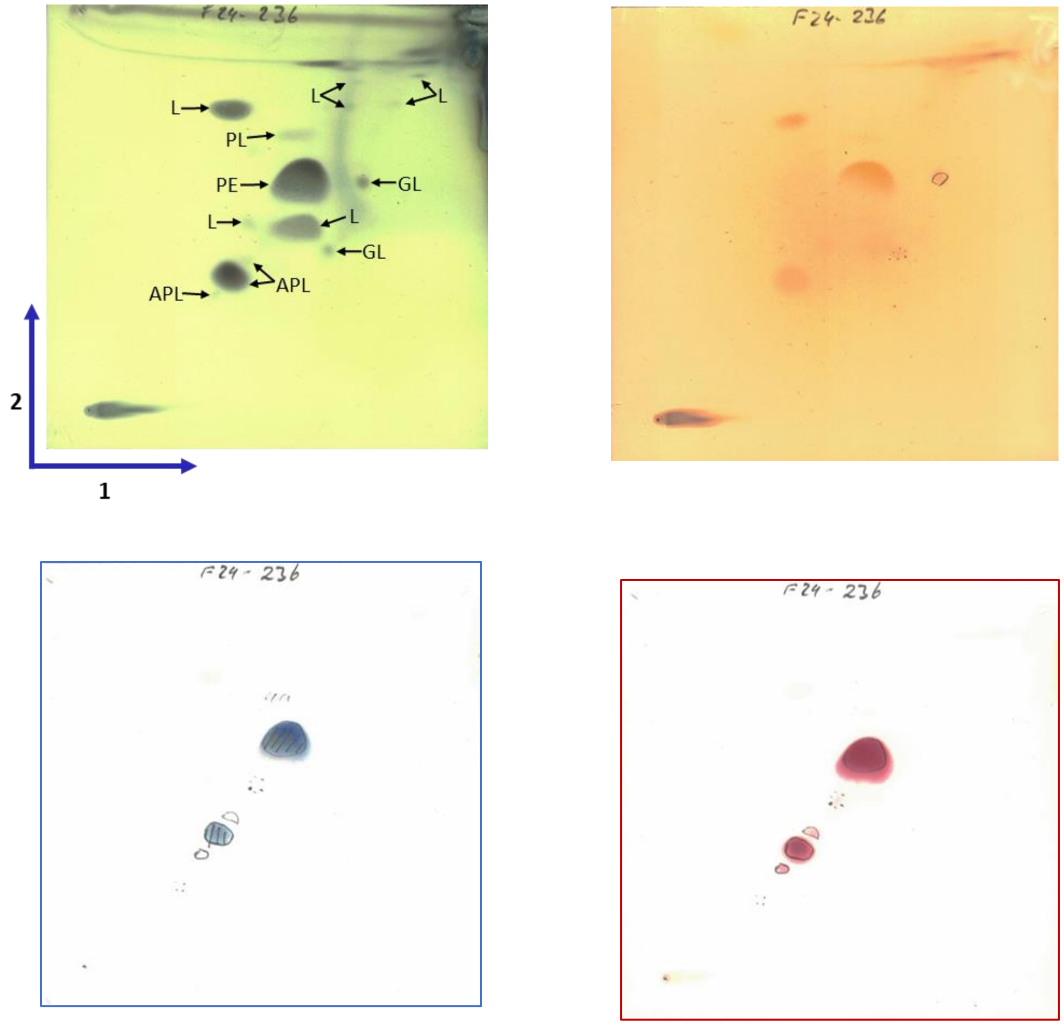

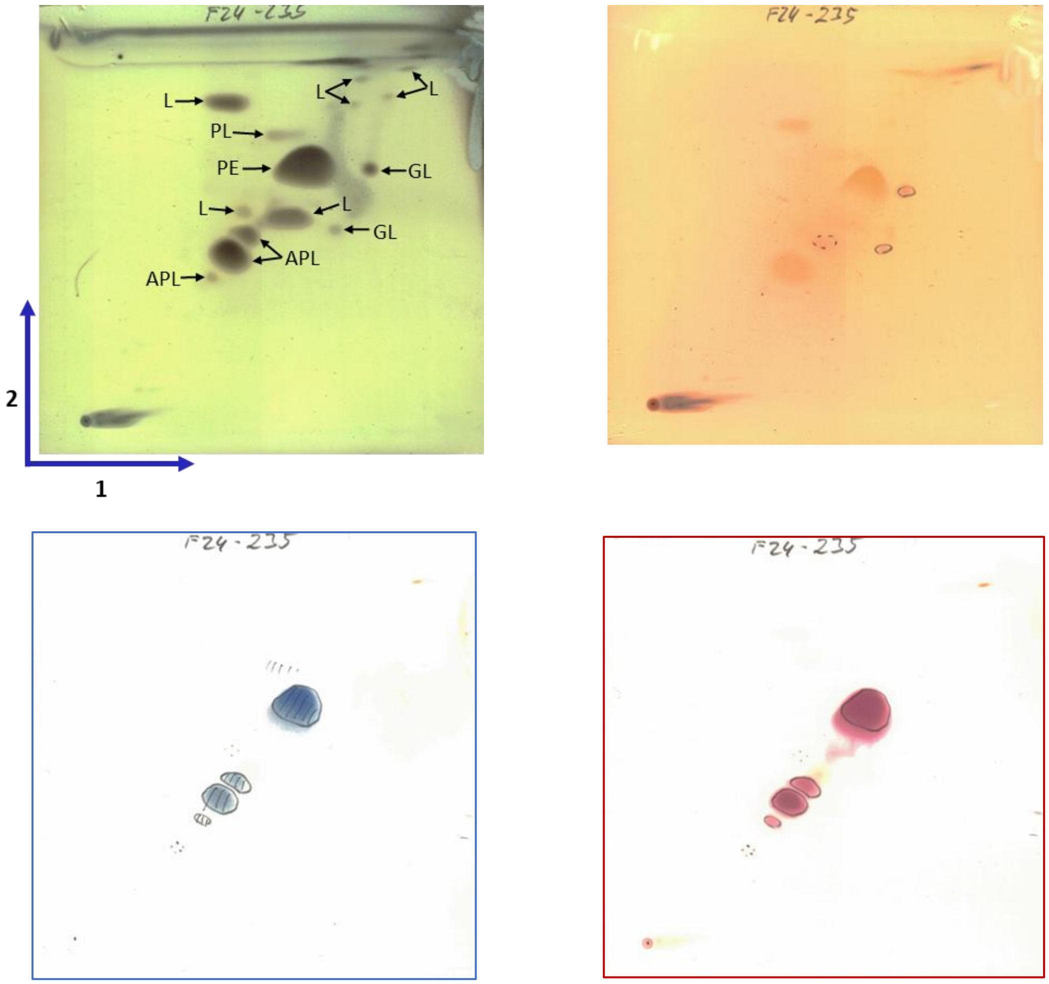
F24-235
L
PL
PE
GL
APL
2
1

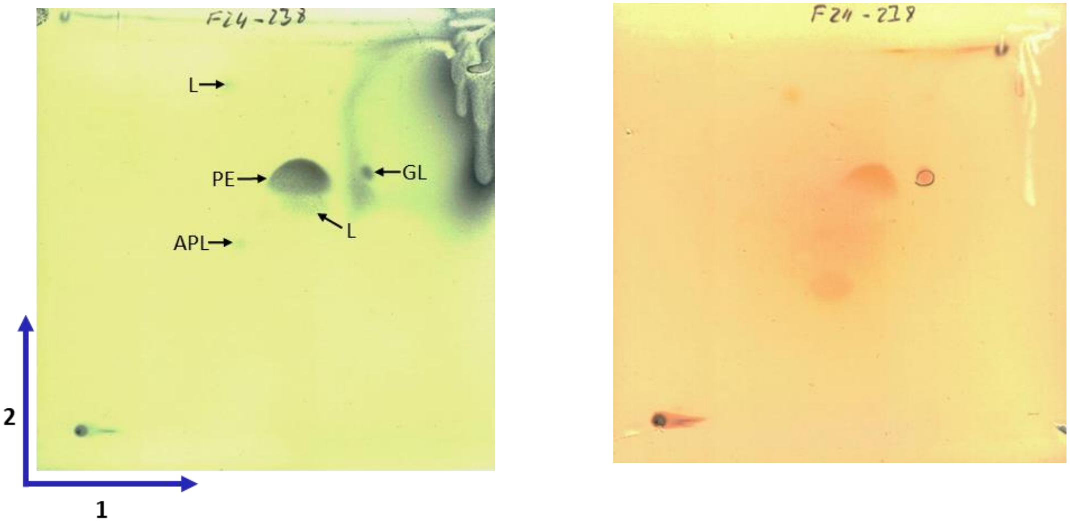
F24-238
L
PE
GL
APL
2
1

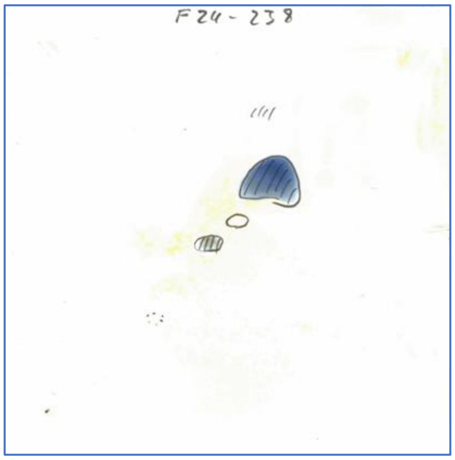
F24-238

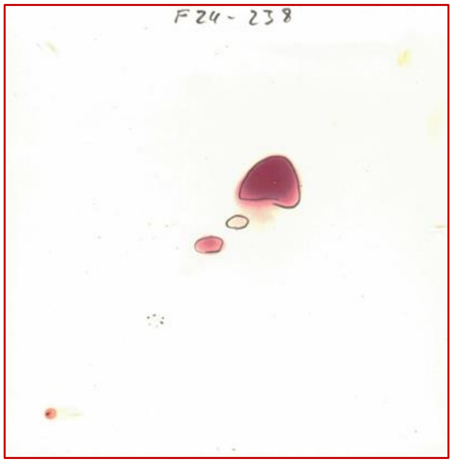
F24-238

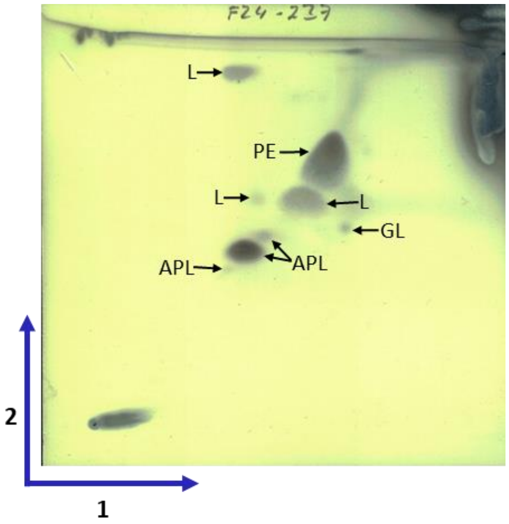
F24-237
L
PE
L
L
GL
APL
APL
2
1

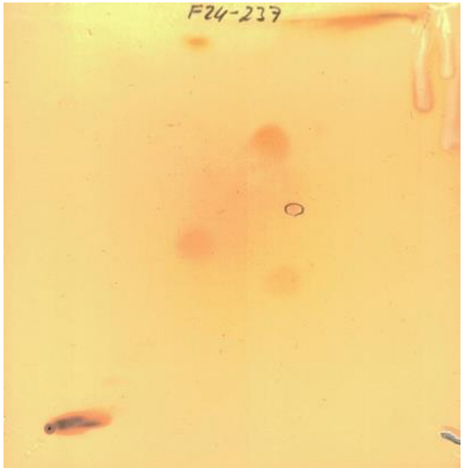
F24-237

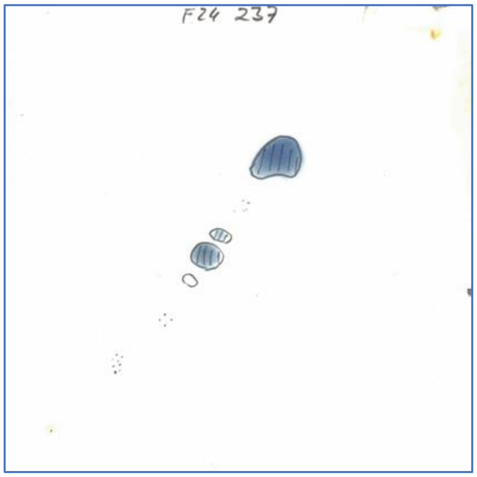
F24 237

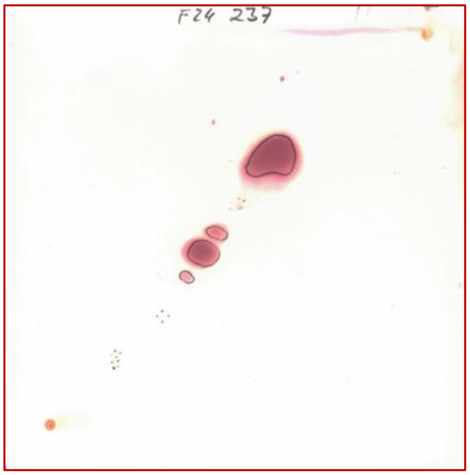
F24 237

Fig. S4 Reconstruction of the phylogenetic position of the investigated strains and related strains (same selection as used in Fig. 1) based on almost full-length 16S rRNA gene sequences (1331 alignment positions). Shown is a neighbour-joining midpoint rooted tree. Bootstrap values ≥70 % are indicated.

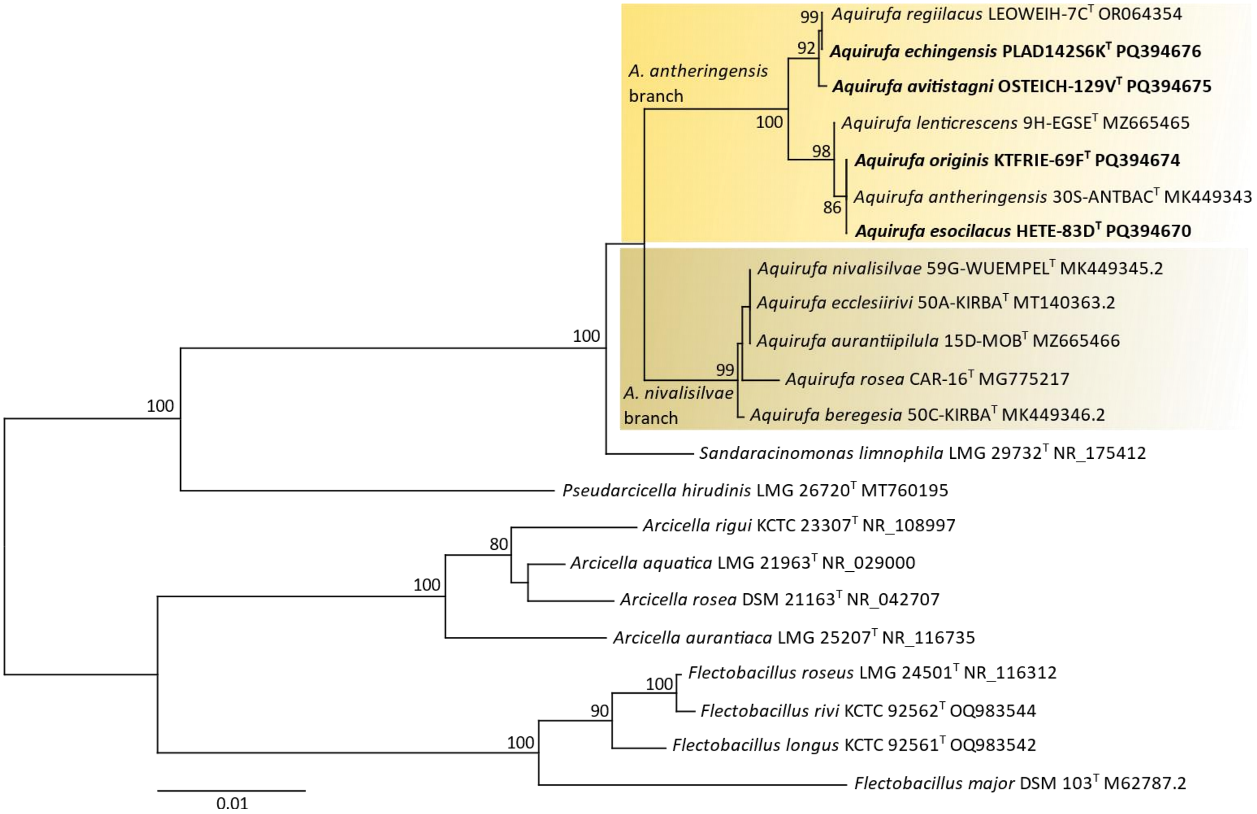

References:

Chen IMA, Chu K, Palaniappan K et al (2019) IMG/M v.5.0: an integrated data management and comparative analysis system for microbial genomes and microbiomes. Nucleic Acids Res 47:D666-D677. https://doi.org/10.1093/nar/gky901

Liu S, Wang H, Chen L et al (2020) Comammox *Nitrospira* within the Yangtze River continuum: community, biogeography, and ecological drivers. The ISME Journal 14:2488-2504. https://doi.org/10.1038/s41396-020-0701-8

Lu J, Gu J, Han J et al (2023) Evaluation of spatiotemporal patterns and water quality conditions using multivariate statistical analysis in the Yangtze River, China. Water 15:3242

Shen M, Li Q, Ren M et al (2019) Trophic status is associated with community structure and metabolic potential of planktonic microbiota in plateau lakes. Front Microbiol 10. https://doi.org/10.3389/fmicb.2019.02560

Shim MJ, Yoon SC, Yoon YY (2018) The influence of dam construction on water quality in the lower Geum River, Korea. Environ Qual Manage 28:113-121. https://doi.org/10.1002/tqem.21591

Yang Y, Huang X, Zhu X et al (2020) Spatiotemporal characteristics of the water quality in the Jinsha River Basin (Panzhihua, China). Water Supply 21:189-203. https://doi.org/10.2166/ws.2020.258

Zhao L, Li W, Lin L et al (2019) Field investigation on river hydrochemical characteristics and larval and juvenile fish in the source region of the Yangtze River. Water 11:1342
